# Improving the performance of ASA in the DAC of 2,5-DMF and ethylene†

**DOI:** 10.1039/d3cy01224g

**Published:** 2023-11-13

**Authors:** Ferdy J. A. G. Coumans, Aleksei Bolshakov, Rim C. J. van de Poll, Dimitra Anastasiadou, Brahim Mezari, Emiel J. M. Hensen

**Affiliations:** a Laboratory of Inorganic Chemistry and Catalysis, Department of Chemical Engineering and Chemistry, Eindhoven University of Technology PO Box 513 5600 MB Eindhoven The Netherlands e.j.m.hensen@tue.nl

## Abstract

A variety of methods are employed to synthesize amorphous silica–alumina (ASA) to resolve the role of Al speciation and surface area in the catalytic performance in the Diels–Alder cycloaddition reaction of 2,5-dimethylfuran and ethylene to *p*-xylene. ASA was prepared by homogeneous deposition–precipitation (HDP) of Al^3+^ on ordered mesoporous silica, *i.e.*, SBA-15 and OMS prepared under hydrothermal synthesis conditions using an imidazole-based template, and one-step flame spray pyrolysis (FSP). IR spectroscopy and ^27^Al MAS NMR showed that the resulting ASA represented a set of materials with distinct textural and acidic properties. ASA prepared by grafting Al to ordered mesoporous silica led to a much higher concentration of Brønsted acid sites (BAS). These samples performed much better in the DAC reaction, with *p*-xylene yields higher than those obtained with a HBeta zeolite benchmark. Materials with Al partially in the bulk of silica (OMS, FSP) and containing significant alumina domains are less acidic and exhibit much lower *p*-xylene yields. These findings point to the importance of Brønsted acidity for *p*-xylene formation. This study shows that careful design of the Al speciation can lead to amorphous silica–alumina with similar DAC performance to microporous zeolites.

## Abstract 2

A variety of methods are employed to synthesize amorphous silica–alumina (ASA) to resolve the role of Al speciation and surface area in the catalytic performance in the Diels–Alder cycloaddition reaction of 2,5-dimethylfuran and ethylene to *p*-xylene.

## Graphical abstract

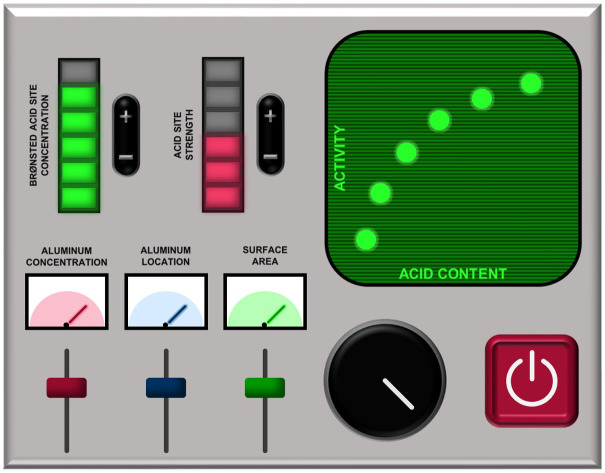

## Introduction

In recent years, the Diels–Alder cycloaddition (DAC) of 2,5-dimethylfuran (2,5-DMF) and ethylene has been explored to produce renewable *p*-xylene.^1–3^ The primary use of *p*-xylene is in manufacturing terephthalic acid and polyethylene terephthalate (PET).^4^ A benefit of replacing the fossil-based route with the more sustainable DAC alternative is the high selectivity to *p*-xylene compared to conventional production procedures.^5–7^ This eliminates the need for the costly isomerization and separation of other isomers (*i.e.*, *ortho*-, *meta*-xylene, and ethylbenzene), usually obtained with catalytic reforming or steam cracking.^8^

Solid Brønsted acids are preferred catalysts for the DAC reaction of 2,5-DMF and ethylene.^7,9–16^ Although the exact nature of the active sites in the reaction mechanism has not been resolved yet, density functional theory (DFT) calculations demonstrated that Brønsted acid sites are the active sites for the dehydration of the oxanorbornene intermediate obtained during the initial addition of ethylene to 2,5-DMF.^17–20^ Microporous materials such as zeolites are among the most investigated catalysts due to their unique properties, which include well-defined and strong acid sites in a potentially beneficial confined space.^17–22^ Amorphous silica–alumina (ASA) also catalyses the DAC reaction; however, its weaker acidity than that of zeolites typically results in lower activity.^9,23,24^ Different views exist on the nature of Brønsted acidity in ASA. Some argue that BAS of zeolitic strength are due to Al substitutions in the silica framework.^25^ In contrast, others underlined the role of penta-coordinated Al in rendering adjacent silanol groups acidic.^26–31^ The number of strong BAS is generally low compared to that in zeolites, resulting from the heterogeneous distribution of Al in ASA. In line with this, it has been emphasized that ASA contains different types of BAS varying in strength.^25^

ASA can be prepared in various manners, such as co-precipitation, deposition–precipitation, hydrothermal synthesis, and flame spray pyrolysis. Homogeneous deposition–precipitation (HDP) stands out as the Al speciation can be better controlled than in the other preparation methods.^32^ HDP involves the grafting and deposition of Al-aqua complexes with silanol groups of the silica support (or the reverse grafting of Si complexes on alumina).^33^ The Brønsted acidity can be increased by depositing more Al. However, clustering of Al into inactive alumina-type domains can occur during the deposition stage, especially when all silanol groups are consumed and during subsequent calcination. As such, it can be envisioned that increasing the surface area of silica at a given silanol density can decrease the formation of undesirable alumina domains and improve the Brønsted acidity of ASA prepared by HDP. Flame spray pyrolysis (FSP) has also been used to prepare ASA with a homogeneous distribution of Si and Al in the mixed oxide. Such FSP-prepared samples often contain significant amounts of penta-coordinated Al species, which have been reported to enhance the acidity.^26–31^

Although materials that can surpass the activity observed by typical zeolite benchmarks have already been reported,^34–36^ we sought to systematically compare the effect of different ASA synthesis methods to improve the Brønsted acidity and the activity in the DAC reaction. A first set of ASA was based on layered mesoporous silica (OMS) in which Al was incorporated either during hydrothermal synthesis^37^ or through HDP of aluminum.^25,33,38^ The FSP method was employed to synthesize ASA samples with Si/Al ratios of 10 and 1.^26–31^ Another ASA sample was prepared *via* HDP of Al on SBA-15.^39–41^ The Al environment was investigated by ^27^Al MAS NMR. ICP elemental analysis and XPS were employed to quantify the amount of aluminium in the bulk and close to the surface. The acid properties were characterized by IR spectroscopy of adsorbed CO and pyridine. Finally, the catalytic activity of the ASA in the DAC reaction of 2,5-DMF with ethylene was determined and compared to a HBeta zeolite reference.

## Experimental

### Preparation of materials

#### ASA-FSP

Several amorphous silica–alumina samples were prepared by FSP. For this purpose, aluminium acetylacetonate (Sigma Aldrich, 99.0%) and tetraethyl orthosilicate (Merck, 99.0%) were dissolved in *p*-xylene (Sigma Aldrich, 99.0%) at atomic Si/Al ratios of 1 and 10 according to a method reported before.^26^ FSP synthesis was carried out in a Tethis NPS 10 machine. The filtered solution was transferred to the apparatus by a syringe pump (5 mL min^−1^) and nebulized with O_2_ (5 L min^−1^). The spray was pyrolyzed in a flame maintained by a gaseous feed of CH_4_ : O_2_ (1.5 : 3 L min^−1^). The formed particles were collected on a cooled Hahnemühle GF6 filter. The resulting samples are denoted as ASA-FSP-10 and ASA-FSP-1.

#### OMS

An ordered mesoporous silica was synthesized using the surfactant 1,2-dimethyl-3-hexadecyl-1*H*-imidazol-3-ium bromide (C_16_IMZ).^37^ C_16_IMZ was dissolved in deionized water at room temperature. Sodium silicate (Merck, SiO_2_ 27.0%, Na_2_O 8.0%) was added dropwise to this solution under stirring. The resulting gel was aged for 2 h at room temperature and had a molar composition of 0.2 C_16_IMZ : 2.7 Na_2_O : 10 SiO_2_ : 300 H_2_O. Subsequently, the gel was transferred into a Teflon-lined stainless-steel autoclave and placed in a rotation oven for 6 days at 115 °C (50 rpm). The resulting solid was thoroughly washed with deionized water and dried overnight at 110 °C. Next, the organics were removed by calcination in air for 6 h at 550 °C (1 °C min^−1^). This sample is denoted as OMS. Al-containing OMS-C_16_dMImz was prepared in two ways. The direct synthesis involved addition of an appropriate amount of Al(OH)_3_ (Sigma Aldrich, 99.8%) to the synthesis solution, which had a final composition of 0.2 C_16_IMZ : 2.7 Na_2_O : 0.05 Al_2_O_3_ : 10 SiO_2_ : 300 H_2_O. This sample is denoted as OMS-10. Alternatively, OMS-C_16_dMImz was aluminated by HDP of Al.^24,25,33,38^ For this purpose, a suspension containing OMS-C_16_IMZ (30 g L^−1^), 0.76 M urea (Sigma Aldrich, 99.5%), and the desired amount of Al(NO_3_)_3_·9H_2_O (Merck, purity 99%) was made. The temperature of the mixture was raised to 90 °C while monitoring the pH. Once a pH above 6 was reached, the suspension was cooled with an ice bath. The grafted resulting solid was retrieved by filtration, rinsed with water, and dried overnight at 110 °C. Finally, the material was calcined for 5 h at 500 °C (0.5 °C min^−1^). The obtained solid is denoted as OMS-HDP-10.

#### SBA-15

SBA-15 was prepared by adding 4 g of Pluronic P123 to a mixture of water (30 g) and 2 M HCl solution (120 g).^39–41^ Once P123 was dissolved, 8.5 g of TEOS was added dropwise, and the resulting solution was kept for 20 h at 25 °C. The mixture was subsequently aged for another 24 h at 90 °C. The resulting suspension was filtered, and the solid was washed and dried overnight at 110 °C. The organics were removed *via* calcination in air for 5 h at 500 °C (0.5 °C min^−1^). This sample is denoted as SBA-15. Aluminium was grafted onto SBA-15 using the HDP procedure described above. The resulting sample was calcined in air for 5 h at 500 °C (0.5 °C min^−1^), yielding SBA-15-HDP-10. Except for the ASA-FSP samples, all Al-containing samples were converted to their proton form *via* triple ion exchange with 1 M NH_4_NO_3_ (0.1 g L^−1^, 70 °C, 2 h), followed by calcination at 500 °C for 4 h (0.5 °C min^−1^).

### Characterization

The Al content of the samples was determined by ICP-OES (Spectro CIROS CCD ICP optical emission spectrometer with axial plasma viewings) analysis of the samples dissolved in a 1 : 1 : 1 volumetric mixture of HF (40% in water) : HNO_3_ (60% in water) : H_2_O.

X-ray diffraction (XRD) patterns were recorded on a Bruker D2 Endeavor diffractometer using Cu Kα. Small angle diffractograms were measured in the range of 0.7° < 2*θ* < 7.0° with a step size of 0.004° and a time per step of 1 s. XRD patterns in the 5.0° < 2*θ* < 60.0° range were registered with a step size of 0.02° and a time per step of 1 s.

The surface areas were determined by N_2_ physisorption at −196 °C on a Micromeritics TriStar II instrument. Before the measurement, samples were degassed at 300 °C for 8 h under an N_2_ flow. The mesopore volume (*V*_meso_) was determined using the Barrett–Joyner–Halenda (BJH) method on the adsorption branch of the isotherm.

The Lewis and Brønsted acid sites were quantified by IR spectroscopy using pyridine on a Bruker Vertex 70v spectrometer. All solids were dehydrated under an O_2_ flow (33 vol% in He) at 400 °C for 1 h before recording the initial background spectra at 150 °C under vacuum. After that, pyridine was dosed until the samples were thoroughly saturated with pyridine, followed by outgassing for 1 h to remove excess pyridine. After evacuation, a spectrum was recorded to quantify the total amount of acid sites. The concentration of strong Brønsted and Lewis acid sites was determined at 423 K after evacuation of the material at 400 °C for 1 h. Bands at 1545 cm^−1^ (BAS) and 1455 cm^−1^ (LAS) were quantified using previously reported extinction coefficients (*ε*_BAS, 1545 cm^−1^_ = 1.67 cm μmol^−1^ and *ε*_LAS, 1455 cm^−1^_ = 2.22 cm μmol^−1^).^42^

CO adsorption measurements were performed on a Bruker Vertex 70v spectrometer at −183 °C using liquid nitrogen. Before recording the background, sample wafers were dehydrated at 400 °C under an O_2_ flow (33 vol% in He) for 1 hour. CO was dosed to the cell using a six-way valve and sample loop (50 μl). The amount of adsorbed CO was obtained after subtraction of the background spectrum. An extinction coefficient of 2.6 cm μmol^−1^ was used to quantify the amount of BAS.^25^

The Al speciation was investigated by ^27^Al magic-angle spinning nuclear magnetic resonance (MAS NMR) spectroscopy. Measurements were performed using an 11.7 T Bruker NEO500 NMR spectrometer with a 2.5 mm MAS probe head spinning at 25 kHz. Spectra were recorded with a single pulse sequence with an 18° pulse, a duration of 1 μs, and an interscan delay of 0.5 s. Before ^27^Al MAS NMR measurements, the samples were hydrated in a desiccator.

MQMAS experiments were performed by the use of a three-pulse sequence *p*_1_–*t*_1_–*p*_2_–*τ*–*p*_3_–*t*_2_ for triple-quantum generation and zero-quantum filtering (strong pulses *p*_1_ = 3.4 μs and *p*_2_ = 1.4 μs at a nutation frequency *ν*_1_ = 100 kHz, a soft pulse *p*_3_ = 11 μs at *ν*_1_ = 8 kHz, a filter time *τ* = 20 μs, and an interscan delay 0.2 s

The amount of carbonaceous deposits on the catalyst used in the DAC reaction was determined with a Mettler Toledo TGA/DSC 1 instrument. In a typical analysis, 20 mg of material was heated to 800 °C at a rate of 5 °C min^−1^ in a diluted O_2_ atmosphere (33 vol% in He).

X-ray photoelectron spectroscopy (XPS) measurements were performed on a K-alpha XPS spectrometer (Thermo Scientific) equipped with a monochromatic Al Kα (1486.6 eV) X-ray source. The spot size was 400 μm, and the pass energy was set at 200 eV and 50 eV for survey and high-resolution spectra, respectively. Binding energy calibration was performed by setting the position of the C 1s peak of adventitious sp^3^ carbon to 284.6 eV. Al 2p and Si 2p regions were used to determine the Si/Al ratio as a function of the probing depth. All the spectra were processed in CasaXPS.

### Diels–Alder cycloaddition

The catalytic activity in the DAC reaction of 2,5-DMF and ethylene was investigated in a 100 mL TOP Industrie autoclave equipped with a mechanical stirrer and a pressure control system. Before adding the reaction solution, 0.3 g of sieved catalyst (250–500 μm) was dried under vacuum for 1 h at 200 °C. Upon reaching the desired reaction temperature of 230 °C, 30 mL of 1.0 M 2,5-DMF and 0.03 M *n*-dodecane (internal standard) in *n*-hexane were added *via* an addition funnel. This was followed by pressurizing the system with ethylene to a pressure of 50 bar. The reaction was ended by disconnecting the heat source and carefully releasing the pressure of the reactor. The reaction mixture was separated from the catalyst by filtration and analysed using GC-FID (Shimadzu GC-FID GC-17A equipped with a Rxi-5 MS column). The 2,5-DMF conversion and product yields were calculated below.

I Conversion (%) = ((*C*_2,5-DMF, *t*=0_ − *C*_2,5-DMF, *t*=*x*_)/*C*_2,5-DMF, *t*=0_)·100%

II Yield_product *i*_ (%) = (*C*_product *i*, *t*=*x*_/*C*_2,5-DMF, *t*=0_)·100%

## Results and discussion

**Fig. 1:**
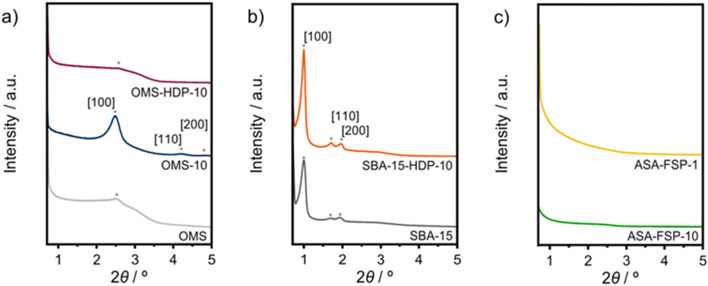
Small-angle XRD diffractograms of the a) OMS, b) SBA-15, and c) FSP samples.

In this work, ASA samples were synthesized using various methods. Table 1 reports the main textural properties of the materials obtained after calcination. The parent OMS and SBA-15 have high surface areas close to 1000 m^2^ g^−1^, which is in line with the literature.^37,43^ The hydrothermally synthesized OMS-10 has a slightly lower surface area than mesoporous silica (OMS). Alumination of SBA-15 by HDP of Al results in a lower surface area and pore volume, although the pore size and wall thickness of SBA-15-HDP-10 are comparable to those of the parent SBA-15 sample. In contrast, the OMS samples contain small pores, according to the BJH analysis. ASA samples prepared by FSP exhibit lower surface areas, with the lowest being obtained at a Si/Al ratio of 1.^26–28^ XRD was used to characterize the calcined samples. The low-angle diffractogram of OMS-10 contains diffraction lines related to layered materials with ordered mesopores arranged in a hexagonal manner (Fig. 1a).^37^ OMS and OMS-HDP-10 have a single broad feature at around 2.6°. The *d*_100_ spacings of the layered structures were calculated to be 34.5 Å for OMS-10, 35.8 Å for OMS, and 33.3 Å for OMS-HDP-10, respectively. As expected for amorphous silica, no diffraction lines were observed at higher 2*θ* angles for the three OMS samples (Fig. S1†). The diffractograms of SBA-15 and SBA-15-HDP-10 shown in Fig. 1b contain the characteristic low-angle diffraction lines associated with the [100], [110], and [200] reflections (2*θ* = 0.99°, 1.72°, and 1.92–1.98°, respectively) of the hexagonal *P*6*mm* symmetry.^39,40^ SBA-15 and SBA-15-HDP-1 have a similar *d*_100_-spacing of 88.9 Å.^39,40^ No reflections were observed in the small-angle regions of the diffractograms of the FSP-prepared ASAs (Fig. 1c). SEM and TEM were used to study the morphology of the samples. The OMS samples were made up of sheets (Fig. S3†).^37^ The typical hexagonal arrangement of cylindrical channels can be observed for SBA-15 and SBA-15-HDP-10, which was not affected by alumination (Fig. S4†). SEM shows that both FSP-ASA samples have a typical morphology for flame spray-synthesized amorphous silica catalysts (Fig. S5†). According to ICP elemental analysis, the Al content of the samples was close to the targeted values (Table 1). However, the final OMS-10 sample contained less aluminium than initially intended.^37^

**Table 1:** Physicochemical properties of the acidic silica-based materials

Sample	Si/Al^a^	Al^a^	*S*_BET_ b	*V*_t_ c	*d*_meso_ d	*a*_0_ e	*w* f
	—	At%	m^2^ g^−1^	cm^3^ g^−1^	nm	nm	nm
OMS	—	—	963	0.65	<2	—	—
OMS-10	17	6	767	0.61	<2	—	—
OMS-HDP-10	10	10	577	0.45	<2	—	—
SBA-15			939	0.96	6.5	10.3	3.8
SBA-15-HDP-10	11	8	486	0.64	6.4	10.3	3.9
ASA-FSP-10	11	9	208	—	—	—	—
ASA-FSP-1	1	45	132	—	—	—	—

a Determined by ICP-OES analysis.

b BET surface area.

c Total pore volume (single point at *p*/*p*° of 0.994).

d Pore size obtained from the BJH analysis model of the adsorption branch.

e *a*_0_ = 2·*d*_100_/
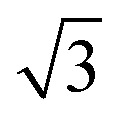
.

f *w* = *a*_0_ − *d*_meso_.

**Fig. 2:**
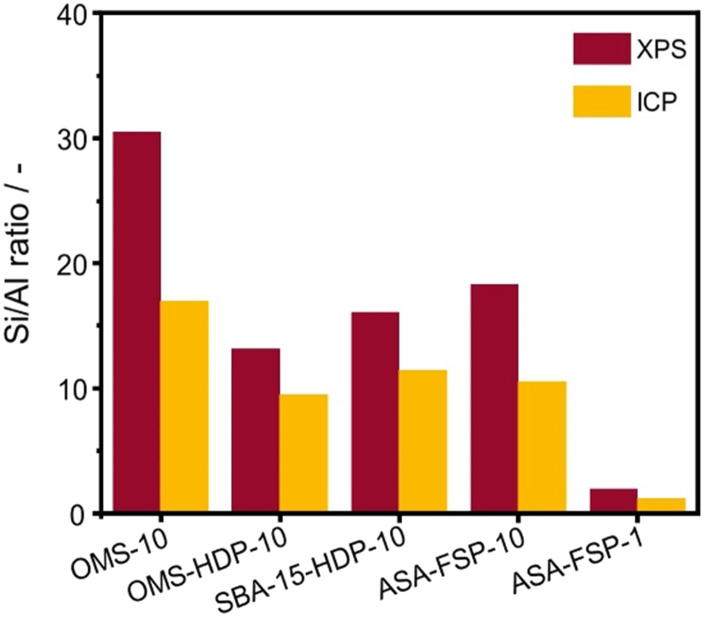
Comparison between bulk and surface composition.

**Fig. 3:**
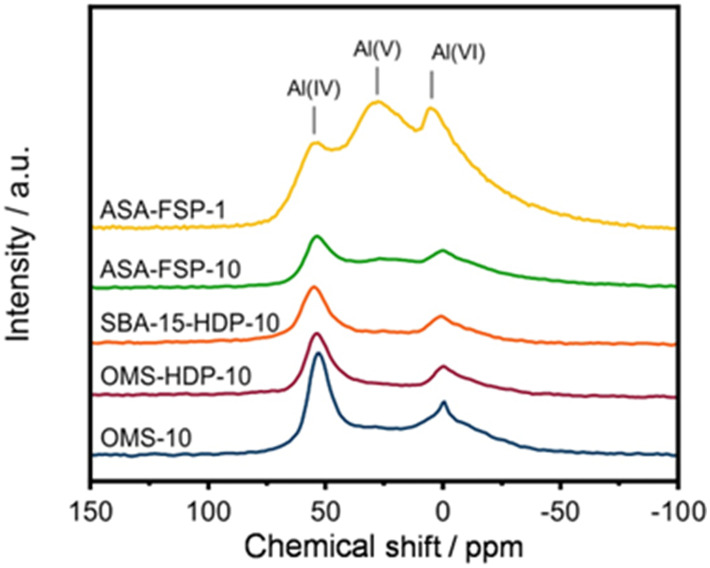
^27^Al MAS NMR spectra of the various ASA samples.

**Fig. 4:**
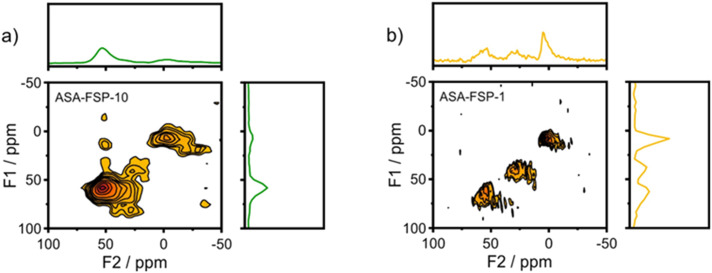
^27^Al MQMAS NMR spectra of a) ASA-FSP-10 and b) ASA-FSP-1.

XPS demonstrates that the surface of the ASA is enriched with aluminium when compared to the bulk composition (Fig. 2). Fig. 3 shows that the OMS-10, OMS-HDP-10, and SBA-15-HDP-10 samples predominantly contain tetrahedral Al species (Al(iv)), as evident from the dominant signal at 53 ppm. There is a small contribution of octahedral Al(vi) at around 0 ppm. Previous work on ASA prepared from silica supports with surface areas of ∼300 m^2^ g^−1^ demonstrated the presence of larger Al(vi) fractions than Al(iv) at similar Al loadings in the ^27^Al MAS NMR spectra.^24^ The presence of octahedral Al could be related to the formation of inactive Al_2_O_3_ domains, similar to the work of Poduval *et al.*^33^ This limits the acidity of the final ASA. The NMR spectra of ASA-FSP-10 and ASA-FSP-1 contain features at 53 ppm of Al(iv) and at 0 ppm due to Al(vi).As also reported in the literature, an additional contribution at ∼27 ppm can be observed in the FSP-1 sample, which can be ascribed to penta-coordinated aluminium (Al(v)).^26–31^^27^Al MQ MAS experiments were performed to gain more detailed information on the aluminium coordination in the FSP samples. The ^27^Al MQ MAS spectrum of ASA-FSP-10 (Fig. 4a) is characterized by very broad signals related to Al(iv) and Al(vi) at around 50 and 0 ppm (F2). The presence of alumina domains is strongly suggested by the interaction between tetrahedral and octahedral aluminium (*e.g.*, the contribution at 50 ppm (F2) and 14 ppm (F1)).^29^ A minor contribution of penta-coordinated Al or distorted Al(iv) species can be discerned at ∼27 ppm (F2). However, most of the Al(v) species arise due to a quadrupolar (horizontal) shift of Al(iv) species. In contrast, the 2D spectrum of ASA-FSP-1 is characterized by three separate peaks on the diagonal line in the MQ MAS spectrum (Fig. 4b). The absence of quadrupolar shifts indicates that aluminium in ASA-FSP-1 has three distinct coordination modes, typical for high Al content ASA.^28^ The ^27^Al MQ MAS spectra of OMS-10, OMS-HDP-10, and SBA-15-HDP-10 are dominated by Al(iv) and minor Al(vi) contributions (Fig. S6†). The narrow signals of OMS-HDP-10 suggest a uniform distribution of Al on the silica support, whereas the peaks in OMS-10 and SBA-15-HDP-10 are broader.

**Fig. 5:**
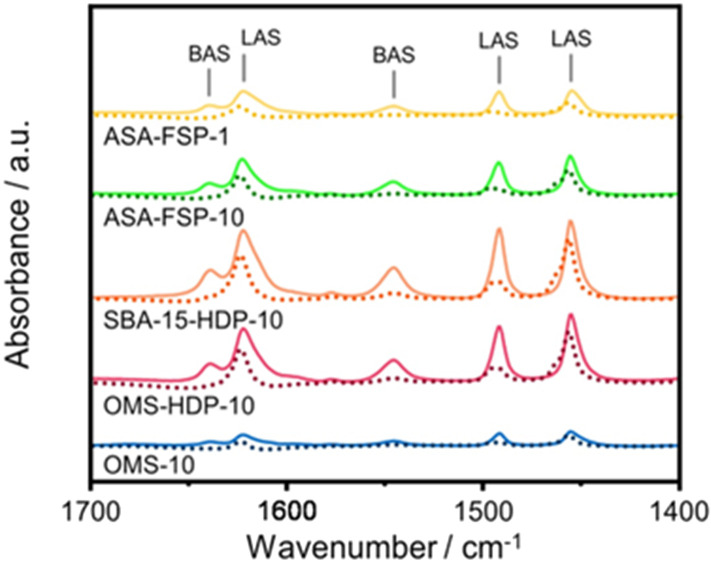
Pyridine adsorption IR spectra of OMS-10, OMS-HPD-10, SBA-15-HDP-10, ASA-FSP-10, and ASA-FSP-1. Bands at 1622, 1491, and 1455 cm^−1^ correspond to Lewis acid sites (LAS), while peaks at 1638 and 1545 cm^−1^ are related to Brønsted acid sites (BAS).

**Fig. 6:**
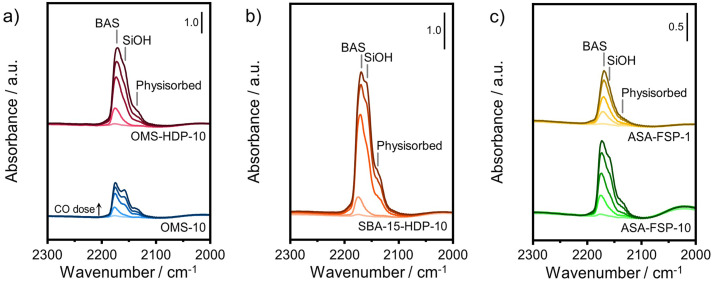
CO IR spectra recorded at liquid nitrogen temperature of a) OMS, b) SBA-15, and c) FSP samples.

**Fig. 7:**
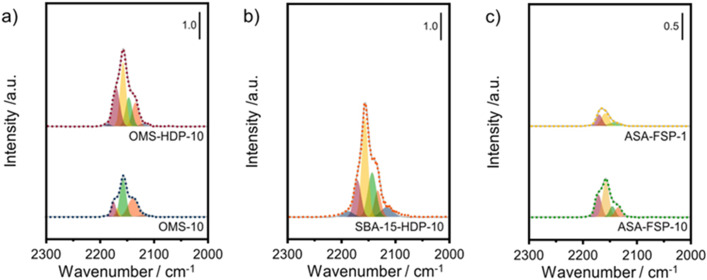
Deconvolution of the CO stretching region of the IR spectra after saturation with CO of the a) OMS, b) SBA-15, and c) FSP samples.

The acidity of the ASA samples was characterized by IR spectroscopy using pyridine and CO as probe molecules. In the pyridine IR spectra, bands related to Lewis acid sites (LAS) can be observed at 1622, 1491, and 1455 cm^−1^ (Fig. 5). The 1638 and 1545 cm^−1^ bands show that the samples also contain Brønsted acid sites (BAS). The numbers of BAS and LAS were estimated by determining the areas of the bands at ∼1545 and ∼1455 cm^−1^ after evacuation at 400 °C, reflecting the total acidities. OMS-10 contains BAS and LAS in amounts of 21 and 41 μmol g^−1^, respectively (Table 2). The BAS and LAS contents of Al-grafted OMS-HDP-10 are much higher, namely 109 and 156 μmol g^−1^. The difference can be explained by a significant fraction of Al in the bulk of OMS-10, as shown by XPS, while most of the Al is at the surface of OMS-HDP-10 (Fig. 2). The BAS and LAS contents of SBA-15-HDP-10 (95 and 133 μmol g^−1^, respectively) are also high, as can be expected for this higher surface area sample to which Al was grafted. Despite its high Al content, the ASA-FSP-1 sample has a relatively low BAS and LAS content of 26 and 36 μmol g^−1^, whereas the ASA-FSP-10 sample with a lower Al content contains more BAS (52 μmol g^−1^) and LAS (67 μmol g^−1^). Upon evacuation at 400 °C, a significant decrease in the intensity of bands related to BAS is observed for all the materials (Fig. 5), indicative of the relatively low acid strength of these sites in ASA.^25^ Of all the BAS, roughly 80–90% are of relatively low strength, as can be judged from Table 2. Despite the relatively large Al(v) content of the ASA-FSP samples, their BAS content is low compared to the other samples. In particular, the BAS content of ASA-FSP-1 is low, which is most likely due to the predominance of alumina domains at the surface. In contrast, considerable amounts of pyridine remain on the LAS after the higher temperature desorption. Deconvolution shows that most LAS are strong, especially OMS-HDP-10 and SBA-15-HDP-10. Fig. 6 shows the CO IR spectra of dehydrated samples as a function of the CO coverage. These spectra were recorded at around −183 °C using liquid nitrogen. A composite band from CO adsorbed on various BAS is observed for all the ASA samples in the 2177–2172 cm^−1^ range, and the emergence of silanol (2165 cm^−1^) and physisorbed CO (2140 cm^−1^) contributions at higher partial pressures.^44^ The IR spectrum of ASA-FSP-1 also contains three small contributions at 2372, 2350, and 2339 cm^−1^ (Fig. S7†), which are commonly related to CO_2_ interactions. This unexpected result was verified in an independent duplo measurement, suggesting the presence of reducible metal oxides in this sample. However, XPS did not reveal the presence of other elements in the ASA-FSP samples. The total BAS concentration was calculated based on the area of the bands in the 2175–2165 cm^−1^ region (Fig. 7). OMS-HDP-10 and SBA-15-HDP-10 have the highest BAS concentration of 147 μmol g^−1^ and 137 μmol g^−1^, respectively. Compared to our previous work,^24^ the use of a silica with high surface area resulted in the formation of more BAS, which can be related to the absence of a major contribution of Al(vi), as confirmed by ^27^Al MAS NMR (Fig. 3). According to CO IR spectroscopy, OMS-10 has a substantially lower BAS concentration of 33 μmol g^−1^ (Table 2) than OMS-HDP-10 due to the high amount of bulk Al. In line with the difference noted in pyridine IR, the BAS concentration of ASA-FSP-1 was only half (19 μmol g^−1^) of the concentration for ASA-FSP-10 (41 μmol g^−1^).

**Table 2:** Physicochemical properties of the various ASA samples

Sample	Pyridine				CO	
	*N*_LAS, total_ a	*N*_LAS, strong_ a	*N*_BAS, total_ a	*N*_BAS_, _strong_^a^	*N*_BAS_ b	*ν*_CO_ c
	μmol g^−1^	μmol g^−1^	μmol g^−1^	μmol g^−1^	μmol g^−1^	cm^−1^
OMS-10	41	27	21	5	33	2174
OMS-HDP-10	156	122	109	18	147	2170
SBA-15-HDP-10	133	107	95	16	137	2170
FSP-10	67	50	52	5	41	2171
FSP-1	36	21	26	1	19	2170
ASA-100^d^	36	27	19	0.6	28	2173
ASA-50^d^	80	63	38	2.0	46	2172
ASA-10^d^	116	72	35	1.8	63	2170
ASA-F10^d^	98	61	33	1.4	58	2170
HBeta-25^d^	134	119	173	73	298	2172

a Concentration of the total Brønsted (1545 cm^−1^) and Lewis (1455 cm^−1^) was obtained after evacuation of pyridine at 150 °C, while the concentration of strong Brønsted was determined after evacuation at 400 °C.

b Amount of Brønsted acid sites obtained after peak fitting of the CO saturated sample.

c IR band position of the CO–OH complex.

d Data were taken from ref. 24.

Previous work demonstrated that ASA prepared by HDP exhibited a lower catalytic performance in the DAC reaction than microporous zeolites, which could be attributed to the formation of alumina domains at higher Al concentrations.^24^ Catalytic activity measurements were performed by adding a 2,5-DMF-containing solution to the dehydrated catalyst when the autoclave reached a temperature of 230 °C. The reaction was then started by pressurizing the reactor to 50 bar with ethylene.

**Fig. 8:**
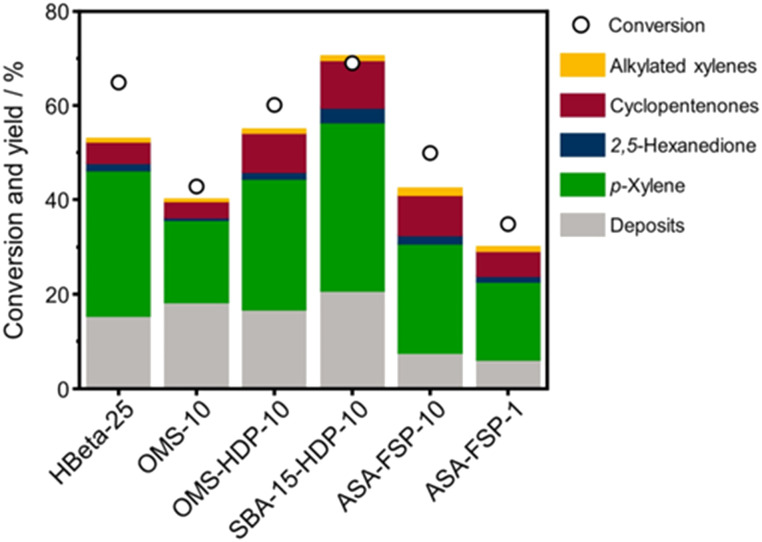
Catalytic performance of ASA catalysts in the Diels–Alder cycloaddition between 2,5-DMF and ethylene. Data on HBeta-25 were taken from the literature.^24^ Conditions: *w*_catalyst_ = 0.3 g, *V* = 0.03 L, *C*_2,5-DMF_ = 1 mol L^−1^, *T* = 230 °C, *p* = 50 bar, *t* = 18 hours.

The 2,5-DMF conversion and product yields are depicted in Fig. 8. The highest conversion is achieved with SBA-15-HDP-10 (69%), followed by the OMS-HDP-10 sample (60%). These two materials exhibit *p*-xylene yields of 36% and 28%, respectively, which compares favourably with the performance of HBeta-25 (2,5-DMF conversion of 65% and *p*-xylene yield of 31%). The OMS-10 sample, in which Al was incorporated during the hydrothermal synthesis, achieved a significantly lower *p*-xylene yield of 17% at a relatively high conversion of 43%. ASA-FSP-10, with a conversion of 50% and a *p*-xylene yield of 23%, performs slightly better than OMS-10. Meanwhile, the performance of ASA-FSP-1 is much lower than that of ASA-FSP-10. All catalysts also produce small amounts of 2,5-hexanedione, cyclopentenones, and alkylated xylenes.

**Fig. 9:**
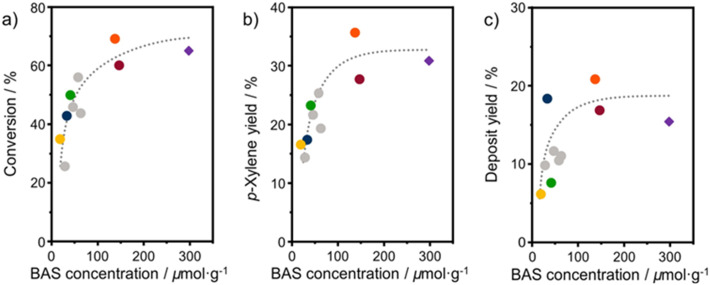
Correlation between BAS concentration (obtained from CO IR) and a) 2,5-DMF conversion; b) *p*-xylene yield; c) deposit yield. Samples: ASA (grey) and HBeta-25 (purple diamond) from a previous study,^24^ OMS-1 (blue), OMS-HDP-10 (red circle), SBA-15-HDP-10 (orange), ASA-FSP-10 (green), and ASA-FSP-1 (yellow). Lines act as a guide to the eye. Conditions: *w*_catalyst_ = 0.3 g, *V* = 0.03 L, *C*_2,5-DMF_ = 1 mol L^−1^, *T* = 230 °C, *p* = 50 bar, *t* = 18 hours.

Turnover frequencies (TOFs) are estimated for the mesoporous ASA materials and compared to that of H-Beta-25 (Table S1†). This comparison underpins the benefit of the open surfaces in the mesoporous catalysts. The finding that some materials present very high TOFs is likely due to the low number of BAS. Overall, these data emphasize that the DAC reaction can be hampered by organic deposits on the surface.^24^ It is likely that the stronger interaction of micropores with heavy compounds will more profoundly limit access of reactants to the acid sites than for the catalysts with predominant mesopores. Fig. 9 shows the 2,5-DMF conversion, *p*-xylene yield, and deposit yield as a function of the BAS concentration. While the conversion initially increases linearly with the BAS concentration, it levels off at higher BAS concentration. Although HBeta-25 has nearly double the amount of BAS, it will reach nearly the same conversion as OMS-HDP-10 and ASA-15-HDP-10, indicating the benefit of placement of BAS in mesopores instead of micropores. Similar trends are observed for the correlations of the *p*-xylene and deposit yields with the BAS concentration (Fig. 9).

The yields increase with the acid site content at lower levels but appear to reach equilibrium at higher BAS concentrations. However, small deviations in the correlation of the deposit can be observed with the OMS-10 and ASA-FSP samples. As discussed, catalyst deactivation is due to the deposition of heavy reaction intermediates on the catalyst surface.^24^ These deposits are most likely formed due to condensation reactions, substantiated by the solutions turning yellowish/brownish during the reaction. We noted that the solution for the experiment with HBeta-25 was darker than for the others which can be attributed to the low adsorption of formed oligomers in the already blocked micropores. This can explain the relatively large amount of unaccounted products observed in the reaction with HBeta-25. Compared to the ASA samples, the external (mesopore) surface area of the zeolite catalyst is much smaller. Therefore, fewer oligomers remain in the solution, especially the ASA sample with the highest surface area, *i.e.*, SBA-15-HDP-10. This results in a mass balance that is more closed when deposits are included, as shown in Fig. 8. In line with this, the mass balances for the experiments with ASA derived from FSP with a much lower surface area are not closed, as also observed for HBeta-25. Although not investigated in this work, previous studies have shown that ASA prepared by the grafting of Al shows higher stability in the DAC reaction compared to the zeolite benchmark.^24^ This has been linked to the absence of Al leaching from the ASA surface and the poor structural integrity of the HBeta crystal upon consecutive reuse.^24,33^

## Conclusions

A set of ASA samples prepared by different methods including templated synthesis to direct hydrothermal synthesis (OMS-10), homogeneous deposition–precipitation of aluminium (OMS-HDP-10 and SBA-15-HDP-10), and flame spray pyrolysis (ASA-FSP-10 and ASA-FSP-1) were prepared to investigate the effect of surface area and the Al location on the DAC reaction of 2,5-DMF with ethylene to *p*-xylene. Al grafting by HDP on mesoporous silica such as OMS and SBA-15 with high surface areas results in ASA with the highest content of BAS. Dispersing Al throughout the sample, as in one-step synthesized OMS-10 and FSP ASAs, results in lower Brønsted acidity. ^27^Al MAS NMR experiments revealed that the aluminium in the ASA obtained through grafting is almost exclusively tetrahedrally coordinated. Samples prepared by FSP and hydrothermal synthesis contain more significant amounts of octahedrally coordinated Al, which indicates the formation of aluminium domains with weaker and predominant Lewis acidity. Overall, a higher Al dispersion on silica yielded the most active samples for the DAC reaction. The two HDP samples reached similar *p*-xylene yields to the HBeta-25 zeolite reference. Samples with a high amount of penta-coordinated Al, earlier suggested to be key to Brønsted acidity in ASA, exhibited a much lower activity. Differences in the texture of the materials lead to varying amounts of deposits during the long reaction tests. Overall, it is clear that the activity strongly correlates with the BAS concentration. However, catalyst deactivation due to the deposition of heavy by-products on the surface leads to a decrease in the reactivity of ASA for zeolite catalysts. 1D and 2D ^27^Al MAS NMR experiments revealed the influence of the synthesis procedure on Al speciation. The well-defined Al-sites of the grafted ASA were reflected by the small spots in the 2D spectra, whereas the FSP-prepared materials exhibited a broad distribution of aluminium. The catalysts prepared *via* the homogeneous deposition–precipitation method (OMS-HDP-10 and SBA-15-HDP-10) displayed the highest concentration of BAS, followed by OMS-10, and finally, both the FSP samples. Catalytic testing revealed that OMS-HDP-10 and SBA-15-HDP-10, having the highest BAS concentration, can match the performance of the HBeta-25 zeolite. The conversion and product yields were found to increase linearly at low BAS concentrations but appear to reach an equilibrium at higher levels. A similar trend was also observed for the deposit formation. However, the amount was found to be dependent on the porous structure, with micro- and mesoporous materials being able to accommodate the build-up of heavier side products.

## Conflicts of interest

There are no conflicts to declare.

## Supplementary Material

CY-013-D3CY01224G-s001
