# Supplementary material for: Improving the performance of ASA in the DAC of 2,5-DMF and ethylene
Source: Catal Sci Technol. 2023 Nov 13;13(24):6959–67. doi: 10.1039/d3cy01224g (PMC10712418; doi:10.1039/d3cy01224g)
Supplement: CY-013-D3CY01224G-s001 [file CY-013-D3CY01224G-s001.pdf]

Electronic Supplementary Material (ESI) for Catalysis Science & Technology.
This journal is © The Royal Society of Chemistry 2023

Supplementary information

Improving the performance of ASA in the DAC of *2,5*-DMF and ethylene

Ferdy J. A. G. Coumans, Aleksei Bolshakov, Rim van de Pol, Dimitra Anastasiadou, Brahim Mezari, Emiel J. M. Hensen*

Laboratory of Inorganic Chemistry and Catalysis, Department of Chemical Engineering and Chemistry, Eindhoven University of Technology, PO Box 513, 5600 MB Eindhoven, The Netherlands

*Corresponding Author

E-mail: e.j.m.hensen@tue.nl

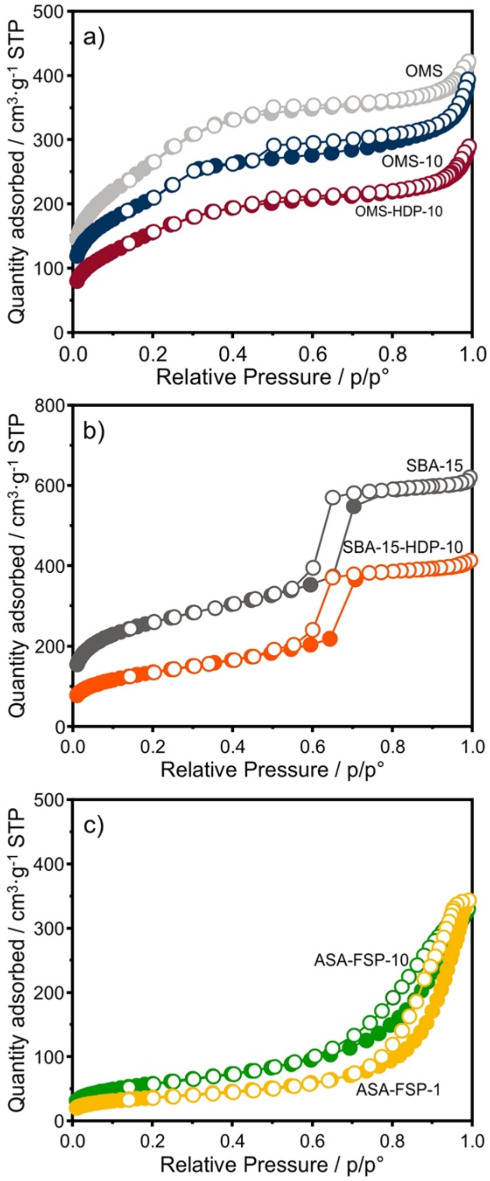

Figure C1. N_2 physisorption adsorption (solid) and desorption (open) isotherms.

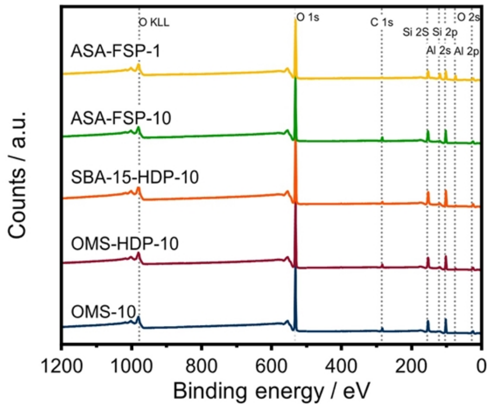

Figure C2. XPS survey spectrum of the various ASA.

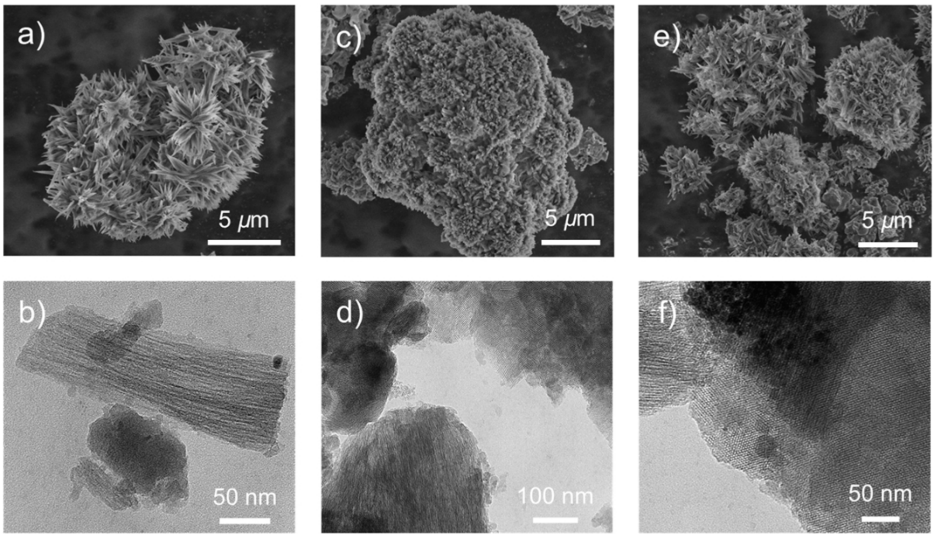

Figure C3. SEM and TEM images of a,b) OMS, c,d) OMS-10, and e,f) OMS-HDP-10.

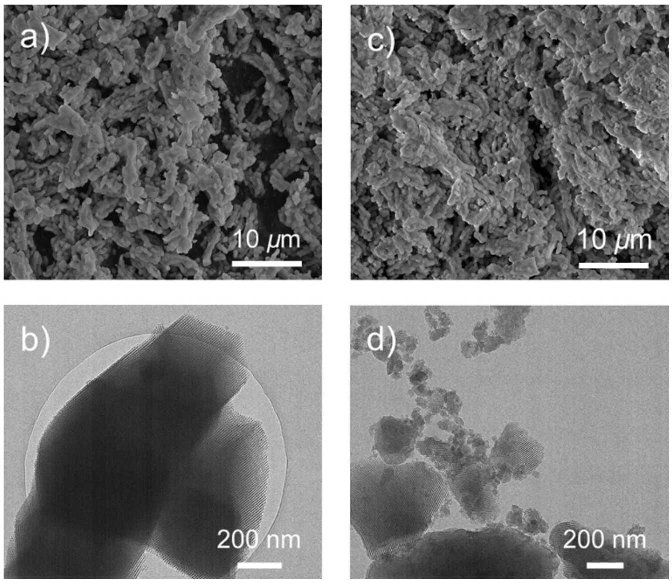

Figure C4. SEM and TEM images of a,b) SBA-15, and c,d) SBA-15-HDP-10.

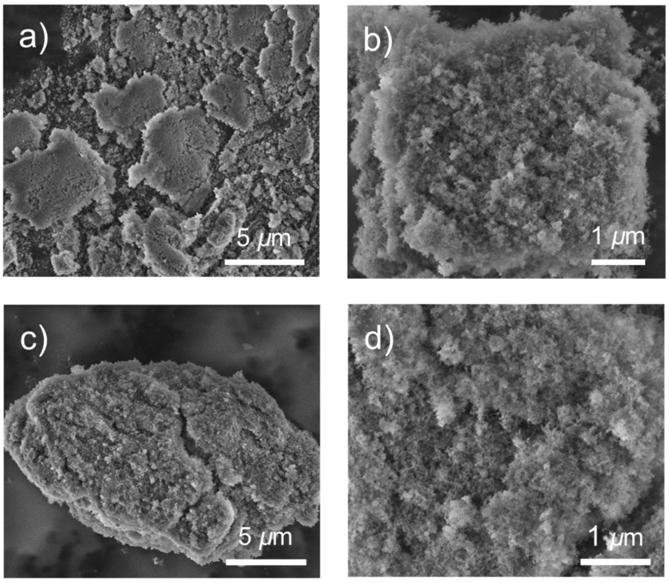

Figure C5. SEM and TEM images of a,b) ASA-FSP-10, c,d) ASA-FSP-1.

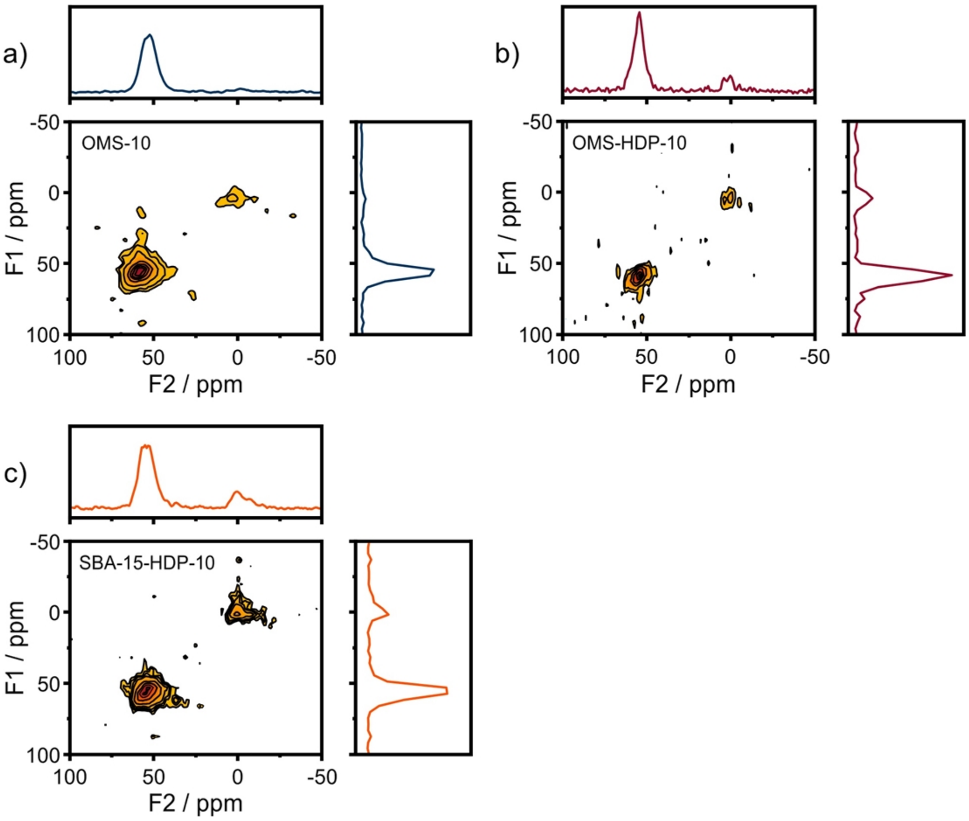

Figure C6. ^{27}Al MQMAS NMR spectra of a) OMS-10, b) OMS-HDP-10, and c) SBA-15-HDP-10. Samples were fully hydrated, and spectra were normalized to the noise.

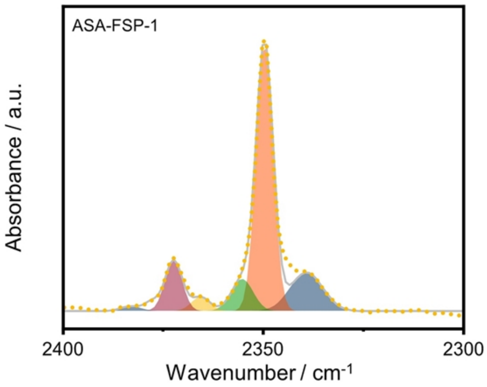

Figure 7. CO IR spectrum of ASA-FSP-1. The presence of these bands has been corroborated in two independent measurements.

Table S1. Turnover frequencies of the investigated catalysts in the DAC reaction of 2,5-DMF with ethylene.

Sample	TOF[a] [$mol_{2,5\text{-DMF, converted}} \cdot mol_{BAS,\ CO}^{-1} \cdot h^{-1}$]
	18 h[b]
ASA-100[c]	52
ASA-50[c]	71
ASA-10[c]	50
ASA-F10[c]	60
ASA-FSP-1	113
ASA-FSP-10	74
OMS-10	79
OMS-HDP-10	24
SBA-15-HDP-10	21
HBeta-25[c]	13

[a] Turnover frequency based on the BAS concentration obtained from the CO adsorption IR experiments [b] reaction time [c] taken from reference[1]

Reference

1. Coumans, F.J.A.G., Demiröz, E., Kosinov, N., and Hensen, E.J.M. (2022). Amorphous Silica-Alumina as Suitable Catalyst for the Diels-Alder Cycloaddition of 2,5-Dimethylfuran and Ethylene to Biobased p-Xylene. ChemCatChem *14*, 1–7. 10.1002/cctc.202200266.
